# Higher serum soluble receptor for advanced glycation end product levels and lower prevalence of metabolic syndrome among Japanese adult men: a cross-sectional study

**DOI:** 10.1186/1758-5996-6-33

**Published:** 2014-03-06

**Authors:** Haruki Momma, Kaijun Niu, Yoritoshi Kobayashi, Cong Huang, Masahiko Chujo, Atsushi Otomo, Hiroko Tadaura, Toshio Miyata, Ryoichi Nagatomi

**Affiliations:** 1 Division of Biomedical Engineering for Health & Welfare, Tohoku University Graduate School of Biomedical Engineering, 2-1 Seiryo-machi, Aoba-ku 980-8575 Sendai, Japan; 2 Department of Epidemiology, School of Public Health, Tianjin Medical University, Tianjin 300070, People’s Republic of China; 3 Department of Medicine and Science in Sports and Exercise, Tohoku University Graduate School of Medicine, Sendai 980-8575, Japan; 4 School of Nursing in Miyagi University, Sendai, Japan; 5 United Centers for Advanced Research and Translational Medicine, Tohoku University Graduate School of Medicine, Sendai 980-8575, Japan

**Keywords:** Endogenous secretory RAGE, Low grade inflammation, CRP

## Abstract

**Background:**

Although several studies showed that decreased soluble receptor for advanced glycation end products (sRAGE) is associated with metabolic syndrome (MetS), inflammation level has not been considered, even though ligand–RAGE interaction induces inflammation. The objective of the study was to determine the association between sRAGE and MetS among Japanese adult in a cross-sectional survey, taking the level of low grade inflammation into consideration.

**Methods:**

Serum soluble RAGE (sRAGE) were measured in 712 men and 176 women aged 30–83 years with serum C-reactive protein (hsCRP) concentration below 3 mg/L. MetS was defined using the criteria of the American Heart Association Scientific Statements of 2009.

**Results:**

After multivariable adjustment, among men, higher sRAGE levels were associated with lower odds of MetS as well as central obesity and elevated blood pressure. Comparing the extreme tertiles of sRAGE, odds ratios (95% confidence interval) were 0.58 (0.36–0.95; *P* for trend = 0.001) for MetS; 0.41 (0.25–0.52; *P* for trend < 0.001) for central obesity; and 0.45 (0.29–0.70; *P* for trend < 0.001) for elevated blood pressure. Moreover, participants were categorized according to their median hsCRP and sRAGE values. Men in the higher hsCRP/higher sRAGE category had a 40% lower odds ratio for MetS than those in the higher hsCRP/lower sRAGE category (*P* = 0.031). Among women, there was no association between sRAGE levels and the prevalence of MetS.

**Conclusions:**

Higher circulating RAGE concentrations were associated with lower prevalence of MetS and its components among Japanese men.

## Introduction

The receptor for advanced glycation end products (RAGE) is a cell surface molecule belonging to the immunoglobulin superfamily that binds many ligands including advanced glycation end products (AGEs) [1]. Circulating forms of RAGE, arising from receptor ectodomain shedding [soluble RAGE (sRAGE)] and secretion of its splice variant [endogenous secretory RAGE (esRAGE)], may competitively inhibit binding of the ligand to membrane-bound RAGE by acting as an endogenous decoy [2]. Indeed, in animal models, recombinant sRAGE administration suppressed the development of atherosclerosis and stabilized established atherosclerosis [3,4]. Thus, circulating RAGE (sRAGE and esRAGE) levels may inversely reflect ligand − RAGE interaction evoked pathogenesis.

Several studies have reported that decreased circulating RAGE concentrations are associated with metabolic syndrome (MetS) risk factors [5-9]. Moreover, participants with MetS showed significantly lower plasma esRAGE concentration than those without MetS [7], and plasma sRAGE levels decreased with increasing number of MetS risk factors [10]. Proinflammatory state [e.g., high concentration of C-reactive protein (CRP)] is considered to have a significant impact on the pathogenesis of MetS and its components [11]. Under low grade chronic inflammation circumstances, higher sRAGE level may attenuate the deteriorating effect of inflammation on MetS and its components. However, such association has not been investigated.

Thus, the purpose of this study was to examine the association between circulating RAGE (sRAGE and esRAGE) concentrations and the prevalence of MetS in Japanese adult in a population-based cross-sectional study, with particular regard to systemic inflammatory level measured by circulating CRP.

## Methods

### Study population

The study participants comprised adult employees enrolled in a prospective study of risk factors for lifestyle-related illnesses or health status at the Sendai Oroshisho Center in Sendai, Japan. The participants received annual health examinations in 2009. This study was conducted during the first week (from Monday to Friday) of August. The details of this study have been described previously [12].

**Figure 1:**
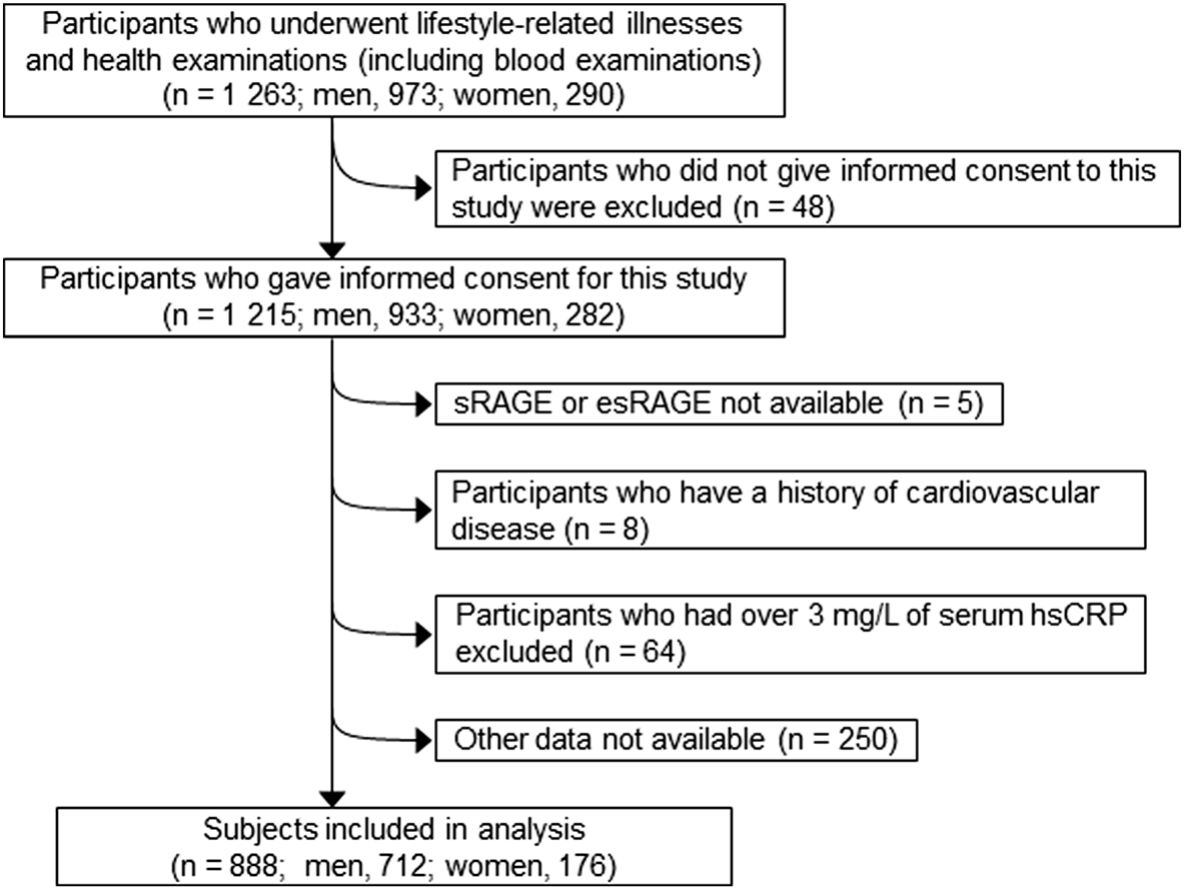
**Flow chart of the sample selection process.** sRAGE, soluble receptor for advanced glycation end products. esRAGE, endogenous secretory RAGE. hsCRP, high sensitivity C-reactive protein.

The sample selection process is described in Figure 1. In 2009, 1 263 participants had undergone health examinations for lifestyle-related illnesses. Of these, 1 215 participated in our survey and provided informed consent for data analysis (response rate, 96.2%). The following participants were excluded: those for whom sRAGE or esRAGE measurements were unavailable (n = 5); those with a history of cardiovascular disease (n = 8); those whose serum CRP concentration was ≥ 3.0 mg/L (n = 64), because people with above 3 mg/L of CRP are considered as being in high risk for cardiovascular disease [13]; and those for whom complete data was not available (n = 250). Thus, 712 men and 176 women were included in the present study. The protocol of this study was approved by the Institutional Review Board of the Tohoku University Graduate School of Medicine.

### Assessment of MetS

Fasting blood samples were drawn from the antecubital vein with minimal tourniquet use, with the participants in a seated position. Samples were collected in siliconized vacuum glass tubes containing sodium fluoride for fasting blood glucose (FBG) analysis, and with no additives for lipid analysis. The FBG concentration was measured enzymatically (Eerotec Co., Ltd., Tokyo, Japan). The concentrations of triglycerides (TG), low-density lipoprotein cholesterol (LDL-C), and high-density lipoprotein cholesterol (HDL-C) were also measured by enzymatic methods using appropriate kits (Sekisui Medical Co., Ltd., Tokyo, Japan). Blood pressure (BP) was measured twice from the upper left arm by means of an automatic device (Yamasu 605P; Kenzmedico, Saitama, Japan) after the participants had rested for 5 min in a sitting position and the mean of the 2 measurements was taken as the BP value. Waist circumference (WC) was measured at the umbilical level with the participants in a standing position and breathing normally. The criteria of the American Heart Association Scientific Statements of 2009 were used to define MetS [14]. The participants were considered to have MetS if they presented ≥3 of the following risk factors: (1) central obesity (≥ 90 cm for men and ≥ 80 cm for women) (2) elevated TG (≥150 mg/dL); (3) reduced HDL-C (< 40 mg/dL for men and < 50 mg/dL for women); (4) elevated BP [systolic BP (SBP) ≥130 mm Hg or diastolic BP (DBP) ≥85 mm Hg]); and (5) elevated FBG (≥100 mg/dL). Those participants who were receiving drug treatment for a given risk factor were considered as having that risk factor.

### Measurements of sRAGE and esRAGE

Serum sRAGE and esRAGE were measured using a commercially available Quantikine ELISA kit (R&D System, Inc., Minneapolis, MN) and the ELISA kit from B-Bridge International (Sunnyvale, CA), respectively. The intra- and interassay coefficients of variation were 2.8% and 6.4%, respectively, for sRAGE and 6.8% and 6.0%, respectively, for esRAGE. Although our hypothesis was that higher serum sRAGE and esRAGE levels were associated with lower prevalence of MetS, previous studies reported conflicting findings for the role of circulating soluble for of RAGE. Thus, because we postulated the possibility that too high or too low serum sRAGE and esRAGE might have an unfavorable influence on the prevalence of MetS and its components resulting in an U or J curve relationship, serum sRAGE and esRAGE concentrations were divided into low, middle, and high tertiles in this study.

### Assessment of other variables

High sensitivity CRP (hsCRP) concentration was determined using N-latex CRP-2 (Siemens Healthcare Japan, Tokyo, Japan). The measurement limit of hsCRP was 0.02 mg/L, and an hsCRP value less than the measurement limit was considered to be 0.01 mg/L. Estimated glomerular filtration rate (eGFR) was calculated using the following equation established by the Japanese Society of Nephrology for Japanese subjects: eGFR (mL/min/1.73 m^2^) = 194 × [serum creatinine (mg/dL)] ^− 1.094^ × (age) ^− 0.287^[15].

Anthropometric parameters (height and body weight) were recorded using a standard protocol. Body mass index (BMI) was calculated as weight (kg) divided by height squared (m^2^).

Age, educational level (<college or ≥ college), occupation (desk-based or not), marital status (married or unmarried), smoking status (never, former, or current) and sleep duration (6–8 h or <6 and >8 h) were obtained through a self-reported questionnaire survey. Daily physical activity (PA) levels were estimated using the International Physical Activity Questionnaire (Japanese version) [16] and divided into three categories [<1.0, 1–22.9, or ≥23.0 metabolic equivalent of tasks (METs) × hours/week] [17]. Total energy intake and alcohol drinking were estimated using a brief self-administered dietary history questionnaire [18]. Alcohol drinking status was categorized into 4 groups (never, ≤3 day(s)/week, 4–6 days/week, or every day). Depressive symptoms were assessed according to the Japanese version of the Self-Rating Depression Scale (SDS) [19]. The participants were considered as depressed when the SDS score was ≥40 [20].

### Statistical analysis

All statistical analyses were performed using SPSS 17.0 for Windows (SPSS, Inc., Chicago, IL, USA).

The distributions of all continuous variables in this study were skewed positively; therefore, we normalized by log-transforming the data in our analyses. Spearman’s rank correlation coefficient was calculated to examine the relationship between sRAGE and esRAGE. To compare the participants’ characteristics, we used the chi-square test and ANOVA for categorical and continuous variables, respectively. Descriptive data are represented as the median (interquartile range) for non-adjusted continuous variables and as percentages for categorical variables. Multiple logistic regression analysis was used to analyze the association of circulating sRAGE or esRAGE concentration with MetS and its components. For analysis, MetS and its components were used as dependent variables, and tertiles of sRAGE or esRAGE were used as independent variables. Analysis was performed after adjustment for potential confounding factors including age, smoking status, drinking status, educational level, occupation, depressive symptoms, PA, total energy consumption, sleeping time, and eGFR (model 1); all parameters in model 1 plus serum hsCRP concentration were used in model 2; all parameters in model 2 plus mutual MetS components were included in model 3 for analysis of MetS components. Moreover, because sex differences existed in the circulating levels of RAGE and hsCRP, and in the prevalence of MetS, we performed separate analyses for men and women. All *P* values for linear trends were calculated using the median values of sRAGE or esRAGE tertiles.

To examine the influence of inflammatory levels on the association between sRAGE and MetS, participants were categorized into higher hsCRP/lower sRAGE, higher hsCRP/higher sRAGE, lower hsCRP/lower sRAGE, or lower hsCRP/higher sRAGE categories according to the median values. Using these categories as independent variables, multiple logistic regression analysis was performed, adjusted for the model 1 and model 4 including all parameters in model 3 except for serum hsCRP concentration. All tests for statistical significance were two-sided and *P* < 0.05 was defined as statistically significant.

## Results

**Figure 2:**
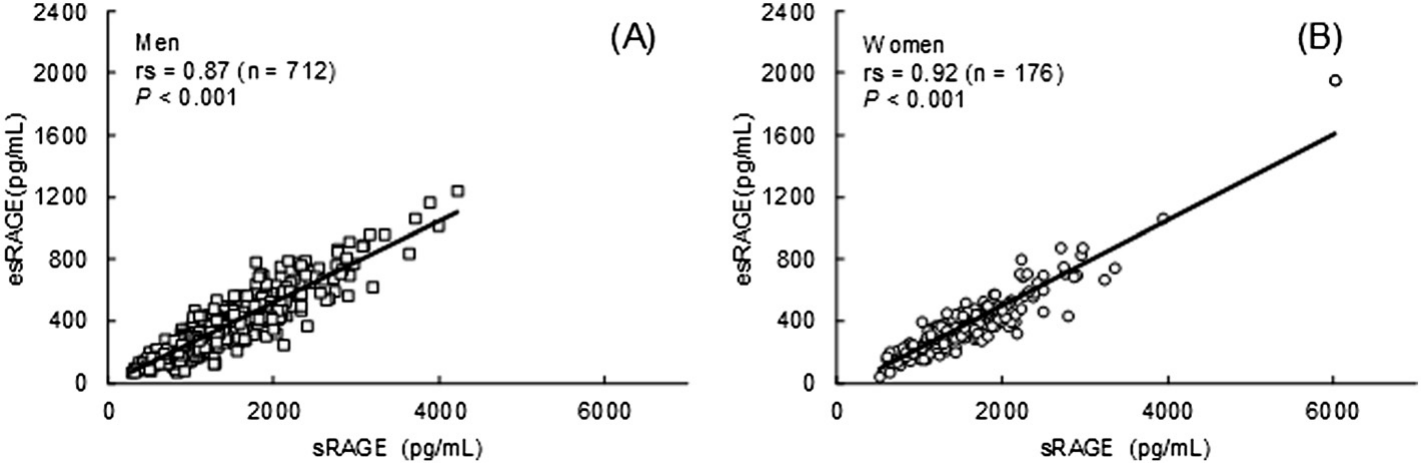
**Relationship between serum sRAGE and esRAGE in men (A) and women (B).** sRAGE, soluble receptor for advanced glycation end products. esRAGE, endogenous secretory RAGE. rs, Spearman rank-correlation coefficient.

Of the 712 men, 179 (25.1%) had ≥3 MetS components. The median (interquartile range) values were 1259.0 (946.4–1609.1) pg/mL for serum sRAGE, 319.0 (235.7–432.2) pg/mL for serum esRAGE, and 0.38 (0.22–0.77) mg/L for hsCRP. Among women, 24 (13.6%) had ≥3 MetS components. The median (interquartile range) values were 1547.9 (1139.6–1922.0) pg/mL for serum sRAGE, 358.1 (259.3–461.5) pg/mL for serum esRAGE, and 0.23 (0.12–0.46) mg/L for hsCRP. The concentration of serum sRAGE was strongly correlated with the concentration of serum esRAGE (Figure 2).

The characteristics according to the tertiles of serum sRAGE in men are presented in Table 1. Serum sRAGE concentration was negatively associated with age, BMI, WC, SBP, DBP, hsCRP (*P* for trend < 0.001), and TG (*P* for trend = 0.036). Moreover, higher tertiles of serum sRAGE had a lower percentage of participants with ≥23 METs × hours/week (*P* for trend = 0.011) and those who drank 7 days/week (*P* for trend < 0.001), and they also had a higher percentage of current smokers (*P* for trend = 0.020). No other significant associations were observed between the groups. Among women, serum sRAGE concentration was negatively associated with BMI, WC, SBP, and hsCRP (Additional file 1: Table S1).

**Table 1:** **Characteristics of the participants according to the tertiles of serum sRAGE in men (n = 712)**^***a***^

	**Tertiles of serum sRAGE**			
**Median, interquartile range (pg/mL)**	**Low (n = 238)**	**Middle (n =237)**	**High (n =237)**	***P *** **for trend**^***b***^
	**(847.6, 710.0–950.0)**	**(1259.1, 1152.8–1377.2)**	**(1835.0, 1608.9–2107.8)**	
Age (years)	51.0 (41.0–57.0)	46.0 (38.0–56.0)	44.0 (38.0–54.0)	<0.001
BMI (kg/m^2^)	24.5 (22.3–26.1)	23.7 (21.7–26.1)	22.7 (20.9–24.4)	<0.001
WC (cm)	86.0 (81.0–92.0)	85.0 (79.0–92.0)	82.0 (77.0–87.0)	<0.001
SBP (mmHg)	133.0 (122.0–143.0)	130.0 (118.0–140.0)	122.0 (114.0–134.0)	<0.001
DBP (mmHg)	84.5 (76.8–91.3)	82.0 (76.0–90.0)	78.0 (70.0–86.0)	<0.001
TG (mg/dL)	125.5 (83.0–174.3)	115.0 (78.0–180.0)	100.0 (73.0–160.0)	0.036
LDL-C (mg/dL)	118.5 (101.8–146.0)	120.0 (101.5–139.5)	118.0 (100.0–137.0)	0.28
HDL-C (mg/dL)	52.0 (43.0–60.3)	51.0 (43.5–61.0)	51.0 (43.0–60.0)	0.34
FBG (mg/dL)	95.0 (90.0–102.3)	94.0 (88.5–103.0)	93.0 (88.0–100.0)	0.07
eGFR (ml/min/1.73 m^2^)	81.3 (73.7–90.4)	81.8 (73.6–90.6)	80.8 (72.4–89.1)	0.16
hsCRP (mg/L)	0.46 (0.27–0.93)	0.40 (0.21–0.78)	0.33 (0.19–0.58)	<0.001
esRAGE (pg/mL)	208.1 (170.3–260.9)	320.4 (278.3–374.6)	480.9 (411.9–573.9)	<0.001
Total energy intake (kcal/day)	1882.0 (1553.8–2309.5)	1866.6 (1523.5–2315.3)	1854.6 (1450.2–2196.5)	0.063
PA				
<1.0 METs · hours/week (%)	20.6	20.7	26.6	0.011
1.0–22.0 METs · hours/week (%)	38.7	42.6	44.3	
≥23.0 METs · hours/week (%)	40.7	36.7	29.1	
Smoking status				
Never smoker (%)	39.5	38.4	32.1	0.020
Former smoker (%)	15.5	10.1	10.5	
Current smoker (%)	45.0	51.5	57.4	
Drinking status				
Non-drinker (%)	12.2	15.6	21.1	<0.001
≤ 3 day(s)/week (%)	29.0	35.4	35.4	
4–6 days/week (%)	21.8	18.2	18.6	
Every day (%)	37.0	30.8	24.9	
Sleep time, ≥6 and ≤8 hours/day (%)	80.3	81.0	76.8	0.35
Education (≥college, %)	34.0	32.9	35.4	0.75
Desk work (%)	80.3	79.3	76.4	0.30
Being married (%)	83.6	78.5	79.3	0.24
Depressive symptoms (SDS ≥40, %)	33.6	28.7	32.1	0.72
Number of MetS components (%)				
No	11.3	20.7	30.4	<0.001
1 component	31.1	26.2	34.2	
2 components	26.9	25.7	18.1	
≥3 components	30.7	27.4	17.3	
Central obesity (%)	33.6	30.8	15.6	<0.001
Elevated BP (%)	71.0	57.4	44.3	<0.001
Elevated FBG (%)	32.8	32.9	25.3	0.078
Elevated TG (%)	35.3	35.4	27.0	0.054
Reduced HDL-C (%)	14.7	13.5	13.9	0.81

^*a*^Data are medians (interquartile range) or proportions. sRAGE, soluble receptor of advanced glycation end-products; BMI, body mass index; WC, Waist circumference; SBP, systolic blood pressure; DBP, diastolic blood pressure; TG, triglyceride; LDL-C, low density lipoprotein cholesterol; HDL-C, high density lipoprotein cholesterol; FBG, fasting blood glucose; eGFR, estimated glomerular filtration rate; hsCRP, high sensitivity C-reactive protein; esRAGE, endogenous secretory RAGE; PA, physical activity; SDS, Self-rating Depression Scale; MetS, metabolic syndrome.

^*b*^Analysis of variance or χ^2^ test.

The prevalence of MetS was 30.7% in the lowest tertile of serum sRAGE, 27.4% in the middle tertile, and 17.3% in highest tertile (*P* for trend = 0.001, Table 1) among men. Moreover, higher serum sRAGE concentration was negatively associated with the number of MetS components (*P* for trend < 0.001), and negatively associated with the prevalence of central obesity and elevated BP (*P* for trend < 0.001). Similar results were obtained for women (Additional file 1: Table S1).

Table 2 shows the relationship of tertiles of serum sRAGE with the prevalence of MetS and its components after adjustment for potential confounders in men. The adjusted odds ratio (95% confidence interval) for MetS in the middle and highest tertiles of serum sRAGE were 0.89 (0.59–1.35) and 0.45 (0.28–0.71) (*P* for trend = 0.001), respectively. Even after adjustment for serum hsCRP concentration, participants with higher serum sRAGE concentration had a lower prevalence of MetS [1.04 (0.67–1.61) for middle tertile, and 0.58 (0.36–0.95) for highest tertile, *P* for trend = 0.038]. Furthermore, participants with higher serum sRAGE concentration had a lower prevalence of central obesity [0.86 (0.58–1.27) for middle tertile, 0.33 (0.21–0.52) for highest tertile, *P* for trend < 0.001], elevated BP [0.61 (0.40–0.93) for middle tertile, 0.38 (0.25–0.57) for highest tertile, *P* for trend < 0.001], and elevated TG [1.00 (0.68–1.48) for middle tertile, and 0.62 (0.41–0.93) for highest tertile, *P* for trend = 0.024], after adjustment for potential confounders. With central obesity and elevated BP, even after adjustment for serum hsCRP concentration (model 2) and mutual MetS components (model 3), the association remained. Moreover, similar results were obtained for the relationships of serum esRAGE concentration with MetS and its components, except for reduced HDL-C in model 1 [0.98 (0.58–1.65) for middle tertile, and 0.44 (0.24–0.79) for highest tertile, *P* for trend = 0.006]. Among women, serum sRAGE level was associated with only elevated BP after adjustment for confounding factors (Additional file 1: Table S2).

**Table 2:** **Relationship of the tertile of serum sRAGE with the prevalence of MetS in men (n = 712)**^***a***^

	**Tertiles of serum sRAGE**			
**Median, interquartile range (pg/mL)**	**Low (n = 238)**	**Middle (n =237)**	**High (n =237)**	***P *** **for trend**^***b***^
	**(847.6, 710.0–950.0)**	**(1259.1, 1152.8–1377.2)**	**(1835.0, 1608.9–2107.8)**	
MetS				
Crude	1.00	0.85 (0.57–1.27)	0.47 (0.31–0.73)	<0.001
Model 1^*c*^	1.00	0.89 (0.59–1.35)	0.45 (0.28–0.71)	0.001
Model 2^*d*^	1.00	1.04 (0.67–1.61)	0.58 (0.36–0.95)	0.038
Central obesity				
Crude	1.00	0.88 (0.60–1.29)	0.37 (0.24–0.57)	<0.001
Model 1^*c*^	1.00	0.86 (0.58–1.27)	0.33 (0.21–0.52)	<0.001
Model 2^*d*^	1.00	0.96 (0.64–1.45)	0.41 (0.25–0.66)	<0.001
Model 3^*e*^	1.00	0.99 (0.65–1.52)	0.46 (0.28–0.75)	0.003
Elevated BP				
Crude	1.00	0.55 (0.38–0.80)	0.33 (0.22–0.48)	<0.001
Model 1^*c*^	1.00	0.61 (0.40–0.93)	0.38 (0.25–0.57)	<0.001
Model 2^*d*^	1.00	0.66 (0.43–1.02)	0.45 (0.29–0.70)	<0.001
Model 3^*e*^	1.00	0.65 (0.42–1.01)	0.49 (0.31–0.77)	0.002
Elevated FBG				
Crude	1.00	1.01 (0.69–1.48)	0.70 (0.47–1.04)	0.078
Model 1^*c*^	1.00	1.17 (0.78–1.75)	0.89 (0.58–1.37)	0.64
Model 2^*d*^	1.00	1.21 (0.81–1.84)	0.97 (0.63–1.51)	0.95
Model 3^*e*^	1.00	1.29 (0.85–1.96)	1.20 (0.76–1.89)	0.40
Elevated TG				
Crude	1.00	1.01 (0.69–1.47)	0.68 (0.46–1.03)	0.054
Model 1^*c*^	1.00	1.00 (0.68–1.48)	0.62 (0.41–0.93)	0.024
Model 2^*d*^	1.00	1.11 (0.74–1.65)	0.75 (0.49–1.65)	0.19
Model 3^*e*^	1.00	1.17 (0.77–1.77)	0.85 (0.54–1.33)	0.50
Reduced HDL-C				
Crude	1.00	0.91 (0.54–1.52)	0.94 (0.56–1.57)	0.81
Model 1^*c*^	1.00	0.77 (0.44–1.33)	0.63 (0.36–1.11)	0.11
Model 2^*d*^	1.00	0.88 (0.45–1.56)	0.87 (0.46–1.56)	0.65
Model 3^*e*^	1.00	0.85 (0.47–1.53)	0.90 (0.48–1.68)	0.74

^*a*^Data are odds (95% confidence interval). sRAGE, soluble receptor of advanced glycation end-products; MetS, metabolic syndrome.

^*b*^Multiple logistic regression analysis.

^*c*^Adjusted for age (continuous variable), smoking status (never, former, or current), drinking status (never, ≤ 3 day(s)/week, 4–6 days/week, or every day), educational level (≥college or not), occupation (desk work or non-desk work), depressive symptoms (Self-Rating Depression Scale ≥40 or not), physical activity (<1.0 METs · hour/week, 1.0–22.9 METs · hour/week, or ≥23.0 METs · hour/week), total energy intake (continuous variable), sleep time (≥6 and ≤8 hours/day or not), and eGFR (continuous variable).

^*d*^Additionally adjusted for serum high sensitivity C-reactive protein concentration (continuous variable).

^*e*^Additionally adjusted for mutual metabolic syndrome components.

We examined the interrelationship between sRAGE, MetS, and hsCRP. Table 3 shows the adjusted odds ratio (95% confidence intervals) for MetS in the higher hsCRP/higher sRAGE, lower hsCRP/lower sRAGE, and lower hsCRP/higher sRAGE categories in men. Participants in the higher hsCRP/higher sRAGE category had a 40% lower odds ratio for MetS than those in the higher hsCRP/lower sRAGE category (*P* = 0.031). Moreover, after adjustment for mutual MetS components (model 2), participants in the higher hsCRP/higher sRAGE category had a 46% lower odds ratio for central obesity (*P* = 0.020) and a 51% lower odds ratio for elevated BP (*P* = 0.007) than those in the higher hsCRP/lower sRAGE category. Among women, there was no difference between higher hsCRP/higher sRAGE and hsCRP/lower sRAGE category after adjustment for confounders (Additional file 1: Table S3).

**Table 3:** **Odds ratios of MetS risk factors by hsCRP and sRAGE categories in men (n = 712)**^***a***^

	**Crude**		**Model 1**^***c***^			
**MetS**	**Odds ratio (95% CI)**	***P***^***b***^	**Odds ratio (95% CI)**	***P***^***b***^		
Higher hsCRP/lower sRAGE (n = 192)	1		1			
Higher hCRP/higher sRAGE (n = 161)	0.65 (0.42–1.01)	0.057	0.60 (0.37–0.95)	0.031		
Lower hsCRP/lower sRAGE (n = 164)	0.26 (0.16–0.43)	<0.001	0.26 (0.15–0.43)	<0.001		
Lower hsCRP/higher sRAGE (n = 195)	0.16 (0.09–0.27)	<0.001	0.16 (0.09–0.28)	<0.001		
	**Crude**		**Model 1**^***c***^		**Model 4**^***d***^	
**Central obesity**	**Odds ratio (95% CI)**	***P***^***b***^	**Odds ratio (95% CI)**	***P***^***b***^	**Odds ratio (95% CI)**	***P***^***b***^
Higher hsCRP/lower sRAGE (n = 192)	1		1		1	
Higher hCRP/higher sRAGE (n = 161)	0.56 (0.36–0.87)	0.010	0.50 (0.31–0.79)	0.003	0.56 (0.35–0.92)	0.020
Lower hsCRP/lower sRAGE (n = 164)	0.38 (0.24–0.61)	<0.001	0.38 (0.23–0.61)	<0.001	0.48 (0.30–0.80)	0.004
Lower hsCRP/higher sRAGE (n = 195)	0.17 (0.10–0.28)	<0.001	0.15 (0.09–0.26)	<0.001	0.21 (0.12–0.37)	<0.001
	**Crude**		**Model 1**^***c***^		**Model 4**^***d***^	
**Elevated BP**	**Odds ratio (95% CI)**	***P***^***b***^	**Odds ratio (95% CI)**	***P***^***b***^	**Odds ratio (95% CI)**	***P***^***b***^
Higher hsCRP/lower sRAGE (n = 192)	1		1		1	
Higher hCRP/higher sRAGE (n = 161)	0.40 (0.26–0.63)	<0.001	0.43 (0.26–0.71)	0.001	0.49 (0.30–0.83)	0.007
Lower hsCRP/lower sRAGE (n = 164)	0.47 (0.30–0.73)	0.001	0.41 (0.25–0.67)	<0.001	0.54 (0.32–0.90)	0.019
Lower hsCRP/higher sRAGE (n = 195)	0.24 (0.16–0.37)	<0.001	0.25 (0.15–0.40)	<0.001	0.35 (0.21–0.58)	<0.001
	**Crude**		**Model 1**^***c***^		**Model 4**^***d***^	
**Elevated FBG**	**Odds ratio (95% CI)**	***P***^***b***^	**Odds ratio (95% CI)**	***P***^***b***^	**Odds ratio (95% CI)**	***P***^***b***^
Higher hsCRP/lower sRAGE (n = 192)	1		1		1	
Higher hCRP/higher sRAGE (n = 161)	0.75 (0.48–1.16)	0.20	0.87 (0.54–1.39)	0.56	1.04 (0.64–1.68)	0.88
Lower hsCRP/lower sRAGE (n = 164)	0.51 (0.32–0.80)	0.004	0.50 (0.31–0.81)	0.005	0.61 (0.37–1.00)	0.050
Lower hsCRP/higher sRAGE (n = 195)	0.47 (0.30–0.73)	0.001	0.60 (0.38–0.96)	0.031	0.86 (0.52–1.42)	0.56
	**Crude**		**Model 1**^***c***^		**Model 4**^***d***^	
**Elevated TG**	**Odds ratio (95% CI)**	***P***^***b***^	**Odds ratio (95% CI)**	***P***^***b***^	**Odds ratio (95% CI)**	***P***^***b***^
Higher hsCRP/lower sRAGE (n = 192)	1		1		1	
Higher hCRP/higher sRAGE (n = 161)	0.91 (0.59–1.39)	0.66	0.86 (0.54–1.32)	0.46	1.01 (0.63–1.63)	0.96
Lower hsCRP/lower sRAGE (n = 164)	0.52 (0.34–0.82)	0.004	0.55 (0.35–0.84)	0.011	0.77 (0.47–1.25)	0.29
Lower hsCRP/higher sRAGE (n = 195)	0.34 (0.21–0.53)	<0.001	0.34 (0.21–0.54)	<0.001	0.53 (0.32–0.89)	0.015
	**Crude**		**Model 1**^***c***^		**Model 4**^***d***^	
**Reduced HDL-C**	**Odds ratio (95% CI)**	***P***^***b***^	**Odds ratio (95% CI)**	***P***^***b***^	**Odds ratio (95% CI)**	***P***^***b***^
Higher hsCRP/lower sRAGE (n = 192)	1		1		1	
Higher hCRP/higher sRAGE (n = 161)	1.09 (0.65–1.82)	0.76	0.78 (0.45–1.38)	0.40	0.79 (0.43–1.47)	0.46
Lower hsCRP/lower sRAGE (n = 164)	0.35 (0.18–0.68)	0.002	0.39 (0.19–0.77)	0.007	0.44 (0.21–0.93)	0.032
Lower hsCRP/higher sRAGE (n = 195)	0.34 (0.18–0.64)	0.001	0.27 (0.14–0.54)	<0.001	0.37 (0.18–0.78)	0.009

^*a*^Participants were categorized by the median values (1259.0 pg/mL for sRAGE; 0.38 mg/L for hsCRP). CI, confidential interval ; sRAGE, soluble receptor of advanced glycation end-products; BP, blood pressure; FBG, fasting blood glucose; TG, triglyceride; HDL-C, high density lipoprotein cholesterol.

^*b*^Multiple logistic regression analysis.

^*c*^Adjusted for age (continuous variable), smoking status (never, former, or current), drinking status (never, ≤ 3 day(s)/week, 4–6 days/week, or every day), educational level (≥college or not), occupation (desk work or non-desk work), depressive symptoms (Self-Rating Depression Scale ≥40 or not), physical activity (<1.0 METs · hour/week, 1.0–22.9 METs · hour/week, or ≥23.0 METs · hour/week), total energy intake (continuous variable), sleep time (≥6 and ≤8 hours/day or not), and eGFR (continuous variable).

^*d*^Additionally adjusted for mutual metabolic syndrome components.

To examine the influence of those excluded because of incomplete data, we examined the associations between circulating sRAGE or esRAGE levels and the prevalence of MetS including those who had incomplete data by complementing the incomplete data. For example, the educational level was categorized into < college, ≥college, or missing value group, and physical activity was categorized into <1.0, 1–22.9, ≥23.0 METs · hours/week, or missing value group. For continuous variables including eGFR and energy intake, missing values were assigned to their medians. In model 2, for men, the adjusted odds ratio (95% confidence interval) for MetS in the middle and highest tertiles of serum sRAGE were 0.83 (0.56–1.26) and 0.46 (0.29–0.72) (*P* for trend = 0.001), respectively. Among women, even inclusion of participants with incomplete data, there was no significant association between serum sRAGE and esRAGE, and MetS. Moreover, for the interrelationship between sRAGE, MetS, and hsCRP among men, participants in the higher hsCRP/higher sRAGE category had a 40% lower odds for MetS than those in the higher hsCRP/lower sRAGE category (0.59 [0.38–0.92], *P* = 0.021).

## Discussion

The present study examined the association between the levels of circulating RAGE (sRAGE and esRAGE) and prevalence of MetS and its component among adult with low grade inflammatory level, through a population-based cross-sectional study. Our results suggested that, among men, after adjustment for serum hsCRP concentration higher circulating RAGE levels were associated with lower prevalence of MetS, central obesity, and elevated BP. Moreover, participants in the higher hsCRP/higher sRAGE category had lower odds ratio of MetS than those in the higher hsCRP/lower sRAGE category. Thus, higher levels of circulating RAGE were associated with lower prevalence of MetS and its components including central obesity and elevated BP among adult men with low grade inflammation.

Previous studies have reported a relationship between circulating RAGE level and MetS [7,10]. For example, Koyama et al. reported that participants with MetS had lower circulating esRAGE concentration than those without it, using a univariate model among type 2 diabetic patients and healthy controls [7]. Recently, in young to middle-aged medication-free non-diabetic subjects, plasma sRAGE concentration was found to decrease as the number of MetS risk factors increased [10]. In the present study, we focused on the relationship between circulating RAGE level and the prevalence of MetS because (i) a proinflammatory state, such as high concentration of circulating CRP, was reported to significantly influence the pathogenesis of MetS and its components [11], and (ii) circulating soluble forms of RAGE may attenuate inflammatory responses via competitive inhibition of ligand-RAGE interaction [2]. However, our finding showed that the significant negative association between serum sRAGE level and the prevalence of MetS remained after adjusting for serum hsCRP level. Therefore, circulating sRAGE as an anti-inflammatory decoy may not be sufficient to explain the contributions of circulating sRAGE to the lower prevalence of MetS. In addition, in the present study, the mean (SD) serum sRAGE concentration was 1344.8 (563.9) pg/ml. Although the concentrations of circulating ligands for RAGE were not measured in this study, a previous study of Japanese adult men reported that the concentration of circulating high mobility group box 1, a RAGE ligand, was 1.69 (0.04) ng/ml [21]. As RAGE is known to be a multi-ligand receptor, the potential ligand concentration likely exceeds the sRAGE concentration. Therefore, it remains unknown whether the concentration of circulating soluble forms of RAGE is sufficient to scavenge accumulating ligands [22].

One of the potential mechanisms explaining the contribution of sRAGE to the decreased incidence of MetS is polymorphisms in RAGE. Kankova et al. [23] demonstrated that subjects bearing the 1704 T allele of the RAGE gene had significantly lower plasma levels of antioxidants, including carotenoids, tocopherol, lutein, and lycopene, than those with the 1704G allele. These results suggest that G1704T is involved in oxidative stress. A recent meta-analysis indicated that the frequency of the 1704 T allele in East Asian populations was 7.76–20.6%, which is substantially higher than that in Caucasians (4.82–9.92%); moreover, the ORs associating the 1704 T allele with diabetes and its complications are higher in East Asian populations [24]. Moreover, RAGE single-nucleotide polymorphism rs2060700 (Gly82Ser) is strongly associated with circulating sRAGE levels [25]. Furthermore, the RAGE Gly82Ser polymorphism is associated with a risk of coronary artery disease [26]. In addition, another RAGE polymorphism might be linked with insulin resistance [27]. Given these results, further comprehensive studies including the evaluation of RAGE polymorphisms are required to clarify the association between circulating soluble forms of RAGE and MetS.

Recently, the role of circulating sRAGE is conflicting. Colhoun et al. investigated the relationship of sRAGE to incident coronary heart disease (CHD) in patients with type 2 diabetes [28]. The patients were followed for 3.9 years, and it was demonstrated that sRAGE concentration were positively associated with incident CHD in type 2 diabetes [28]. Similar results have been obtained among type 1 diabetes [29,30] and elderly women [31]. These results suggest that serum sRAGE concentration has a bivalent role. In conditions of elevated inflammatory level, such as diabetes or aging, serum sRAGE may be a marker of inflammation rather than a decoy, because there is emerging evidence that proteolytic cleavage, through which the component is formed, is part of a regulatory process and may reflect ongoing inflammation [32]. One would, therefore, expect higher concentration to be associated with more vascular disease. In contrast, among our participants, higher sRAGE concentration was associated with a lower prevalence of MetS even if they were in the higher hsCRP category. Consistent with our results, Selvin et al. have demonstrated a negative relationship between low concentration of circulating sRAGE and the risks of diabetes, CHD, and mortality in a community-based prospective cohort of middle-aged adults [33]. Thus, these results suggested that serum sRAGE concentration may be a marker of inflammation in conditions of elevated CRP, whereas they may be negatively associated with a risk of cardiovascular diseases and its risk factors, such as MetS, in conditions of low grade inflammation.

Although the details of regulation of soluble forms of RAGE have not been revealed, it is possible that the risk factors of MetS or cardiovascular diseases might influence the amount of circulating soluble forms of RAGE. Previous studies have reported the relationships between higher serum sRAGE concentration and lower BMI and WC in general population [5] and non-diabetic Japanese populations [6], and consistent with this findings, there was a positive correlation with adiponectin [28]. We also showed that higher sRAGE concentration was associated with lower prevalence of central obesity in this study. Thus, although the mechanism of the association between lower level of circulating soluble forms of RAGE and obesity is not still clear, the impaired function of adipocyte might be contributing to lower level of circulating soluble forms of RAGE. An alternative possibility is an up-regulation of full-length RAGE shedding by treatment of statin. A recent in vitro study showed that reduction of cellular cholesterol by statins significantly increases the levels of soluble RAGE by enhancement of full-length RAGE shedding [34]. This result was supported by clinical studies with hypercholesterolemic [35]. Further experimental studies are necessary to elucidate the regulation of soluble forms of RAGE.

In addition to central obesity, there was a negative association between the prevalence of elevated BP and serum sRAGE levels. Geroldi et al. showed that the plasma concentration of sRAGE was lower in hypertensive subjects than in normotensive controls [9]. Because crosslinking between collagen molecules and AGE could be implicated in the pathogenesis of arterial stiffening and hypertension [36], the secreted form of RAGE could prevent hypertension by binding to circulating AGE, thus preventing them from forming protein–protein crosslinks. A significantly elevated AGE concentration was found in the vascular smooth muscle cells of spontaneously hypertensive rats [37]. Taken together, higher circulating sRAGE concentrations may be associated with lower the prevalence of MetS by lowering the prevalence of central obesity and elevated BP.

There are some limitations in this study. First, because the sample size of our female participants was relatively small, statistical power may not have been sufficient to obtain statistical significance. Whether the abovementioned relationship is present in women populations remains unknown. Second, because this study used a cross-sectional design, we cannot conclude the causal relationship between sRAGE and esRAGE and the prevalence of MetS and its components. A larger population-based prospective study needs to be performed to further confirm the causal relationship between sRAGE and esRAGE and the prevalence of MetS and its components.

## Conclusion

In conclusion, higher concentrations of circulating RAGE were associated with lower prevalence of MetS and its components including central obesity and elevated BP among Japanese adult men with low grade inflammation.

## Abbreviations

sRAGE: Soluble receptor for advanced glycation end products; esRAGE: Endogenous secretory receptor for advanced glycation end products; MetS: Metabolic syndrome; (hs)CRP: (High sensitivity) C-reactive protein; AGEs: Advanced glycation end products; FBG: Fasting blood glucose; TG: Triglycerides; LDL-C: Low-density lipoprotein cholesterol; HDL-C: High-density lipoprotein cholesterol; WC: Waist circumference; BP: Blood pressure; SBP: Systolic blood pressure; DBP: Diastolic blood pressure; eGFR: Estimated glomerular filtration rate; BMI: Body mass index; PA: Physical activity; METs: Metabolic equivalent of tasks; SDS: Self-rating depression scale; ANOVA: Analysis of variance; ANCOVA: Analysis of covariance; CHD: Coronary heart disease.

## Competing interests

The authors declare that they have no competing interests.

## Authors’ contributions

HM and RN conceived the study. HM, RN, KN, and YK designed the study. HM, KN, YK, AO, MC, CH, and HT did the data collection and processing. HM, KN, YK, AO MC, and CH did the statistical analysis. HM and RN wrote the manuscript. HM, TM and RN contributed substantially to the interpretation of results and provided critical revisions to the manuscript. RN took overall responsibility for the integrity of the study. All authors read and approved the final manuscript.

## Supplementary Material

Additional file 1: Table S1 Characteristics of the participants according to the tertiles of serum sRAGE in women (n = 176)^*a*^*.***Table S2.** Relationship of the tertile of serum sRAGE with the prevalence of MetS risk factors in women (n = 176)^*a*^. **Table S3.** Odds ratios of MetS risk factors by hsCRP and sRAGE categories in women (n = 176)^*a*^. Click here for file
