# Supplementary material for: Higher serum soluble receptor for advanced glycation end product levels and lower prevalence of metabolic syndrome among Japanese adult men: a cross-sectional study
Source: Diabetol Metab Syndr. 2014 Mar 6;6:33. doi: 10.1186/1758-5996-6-33 (PMC4016590; doi:10.1186/1758-5996-6-33)
Supplement: Additional file 1: Table S1 — Characteristics of the participants according to the tertiles of serum sRAGE in women (n = 176)a.Table S2. Relationship of the tertile of serum sRAGE with the prevalence of MetS risk factors in women (n = 176)a. Table S3. Odds ratios of MetS risk factors by hsCRP and sRAGE categories in women (n = 176)a. [file 1758-5996-6-33-S1.docx]

Additonal file 1

| **TABLE S1**. Characteristics of the participants according to the tertiles of serum sRAGE in women (n = 176)*^a^* | | | | | |
| --- | --- | --- | --- | --- | --- |
|  | Tertiles of serum sRAGE | | |  | |
| Median, interquartile range (pg/mL) | Low (n = 59)  (1014.7, 831.8–1139.6) | Middle (n = 59)  (1555.6, 14279.6–1678.1) | High (n =58)  (2102.3, 1922.0–2490.6) | | *P* for trend *^b^* |
| Age (years) | 46.0 (37.0–54.0) | 46.0 (39.0–58.0) | 44.0 (36.0–51.0) | | 0.22 |
| BMI (kg/m^2^) | 22.5 (20.7–26.2) | 20.8 (19.8–22.3) | 20.4 (18.5–22.9) | | <0.001 |
| WC (cm) | 81.0 (73.0–88.0) | 78.0 (72.0–83.0) | 75.0 (69.8–80.5) | | 0.001 |
| SBP (mmHg) | 120.0 (110.0–133.0) | 120.0 (110.0–133.0) | 110.0 (102.0–120.0) | | 0.013 |
| DBP (mmHg) | 78.0 (68.0–86.0) | 74.0 (70.0–82.0) | 70.0 (67.5–78.0) | | 0.056 |
| TG (mg/dL) | 71.0 (49.0–98.0) | 69.0 (52.0–93.0) | 63.0 (49.5–76.3) | | 0.26 |
| LDL-C (mg/dL) | 120.0 (96.0–146.0) | 114.0 (95.0–148.0) | 112.5 (97.8–135.8) | | 0.47 |
| HDL-C (mg/dL) | 59.0 (51.0–72.0) | 65.0 (56.0–74.0) | 65.0 (60.0–70.5) | | 0.08 |
| FBG (mg/dL) | 89.0 (83.0–96.0) | 88.0 (84.0–94.0) | 85.5 (81.8–90.3) | | 0.06 |
| eGFR (ml/min/1.73m^2^) | 79.7 (72.0–88.7) | 77.5 (67.2–85.1) | 78.0 (68.4–84.1) | | 0.17 |
| hsCRP (mg/L) | 0.24 (0.14–0.57) | 0.25 (0.12–0.48) | 0.16 (0.09–0.34) | | 0.034 |
| esRAGE (pg/mL) | 231.1 (178.8–288.9) | 354.0 (309.3–442.2) | 492.3 (420.7–689.2) | | <0.001 |
| Total energy intake (kcal/day) | 1439.3 (1225.6–1971.5) | 1608.4 (1224.8–2163.3) | 1436.7 (1194.1–1724.6) | | 0.62 |
| PA |  |  |  | |  |
| <1.0 METs·hours/week (%) | 23.7 | 22.0 | 31.0 | | 0.71 |
| 1.0–22.0 METs·hours/week (%) | 49.2 | 47.5 | 39.7 | |  |
| ≥23.0 METs·hours/week (%) | 27.1 | 30.5 | 29.3 | |  |
| Smoking status |  |  |  | |  |
| Never smoker (%) | 66.1 | 74.6 | 87.9 | | 0.005 |
| Former smoker (%) | 8.5 | 5.1 | 5.2 | |  |
| Current smoker (%) | 25.4 | 20.3 | 6.9 | |  |
| Drinking status |  |  |  | |  |
| Non-drinker (%) | 47.5 | 40.7 | 32.8 | | 0.99 |
| ≤ 3 day(s)/week (%) | 28.8 | 37.3 | 48.3 | |  |
| 4–6 days/week (%) | 10.6 | 3.4 | 15.5 | |  |
| Every day (%) | 13.6 | 18.6 | 3.4 | |  |
| Sleep time, ≥6 and ≤8 hours/day (%) | 83.1 | 66.1 | 74.1 | | 0.27 |
| Education (≥college, %) | 10.2 | 8.5 | 8.6 | | 0.77 |
| Desk work (%) | 84.7 | 91.5 | 94.8 | | 0.07 |
| Being married (%) | 49.2 | 57.6 | 48.3 | | 0.93 |
| Depressive symptoms (SDS ≥40, %) | 52.5 | 61.0 | 60.3 | | 0.39 |
| Number of MetS components |  |  |  | |  |
| No | 27.1 | 39.0 | 62.1 | | <0.001 |
| 1 component | 30.5 | 33.9 | 20.7 | |  |
| 2 components | 20.3 | 16.9 | 8.6 | |  |
| ≥3 components | 22.1 | 10.2 | 8.6 | |  |
| Central Obesity (%) | 52.5 | 35.6 | 27.6 | | 0.006 |
| Elevated BP (%) | 47.5 | 33.9 | 17.2 | | 0.001 |
| Elevated FBG (%) | 15.3 | 8.5 | 5.2 | | 0.066 |
| Elevated TG (%) | 11.9 | 11.9 | 5.2 | | 0.22 |
| Reduced HDL-C (%) | 20.3 | 8.5 | 8.6 | | 0.056 |
| *^a^* Data are medians (interquartile range) or proportions. sRAGE, soluble receptor of advanced glycation end-products; BMI, body mass index; WC, Waist circumference; SBP, systolic blood pressure; DBP, diastolic blood pressure; TG, triglyceride; LDL-C, low density lipoprotein cholesterol; HDL-C, high density lipoprotein cholesterol; FBG, fasting blood glucose; eGFR, estimated glomerular filtration rate; hsCRP, high sensitivity C-reactive protein; esRAGE, endogenous secretory RAGE; PA, physical activity; SDS, Self-rating Depression Scale; MetS, metabolic syndrome.  *^b^*Analysis of variance or χ^2^ test. | | | | | |

| **TABLE S2**. Relationship of the tertile of serum sRAGE with the prevalence of MetS risk factors in women (n = 176) *^a^*. | | | | |
| --- | --- | --- | --- | --- |
|  | Tertiles of serum sRAGE | | |  |
| Median, interquartile range  (pg/mL) | Low (n = 59)  (1014.7, 831.8–1139.6) | Middle (n = 59)  (1555.6, 14279.6–1678.1) | High (n =58)  (2102.3, 1922.0–2490.6) | *P* for trend *^b^* |
| MetS |  |  |  |  |
| Crude | 1.00 | 0.40 (0.14–1.14) | 0.33 (0.11–1.01) | 0.039 |
| Model 1*^c^* | 1.00 | 0.22 (0.06–0.86) | 0.51 (0.13–1.96) | 0.17 |
| Model 2*^d^* | 1.00 | 0.19 (0.04–0.84) | 0.65 (0.15–2.77) | 0.28 |
| Central Obesity |  |  |  |  |
| Crude | 1.00 | 0.50 (0.24–1.04) | 0.34 (0.16–0.74) | 0.006 |
| Model 1*^c^* | 1.00 | 0.44 (0.19–1.01) | 0.31 (0.13–0.76) | 0.009 |
| Model 2*^d^* | 1.00 | 0.44 (0.18–1.06) | 0.35 (0.14–0.91) | 0.028 |
| Model 3*^e^* | 1.00 | 0.52 (0.20–1.35) | 0.38 (0.14–1.07) | 0.065 |
| Elevated BP |  |  |  |  |
| Crude | 1.00 | 0.57 (0.27–1.19) | 0.23 (0.10–0.54) | 0.001 |
| Model 1*^c^* | 1.00 | 0.48 (0.21–1.10) | 0.22 (0.09–0.59) | 0.002 |
| Model 2*^d^* | 1.00 | 0.49 (0.21–1.12) | 0.23 (0.09–0.62) | 0.003 |
| Model 3*^e^* | 1.00 | 0.58 (0.24–1.41) | 0.24 (0.08–0.69) | 0.008 |
| Elevated FBG |  |  |  |  |
| Crude | 1.00 | 0.51 (0.16–1.64) | 0.30 (0.08–1.18) | 0.072 |
| Model 1*^c^* | 1.00 | 0.08 (0.01–0.61) | 0.19 (0.03–1.34) | 0.048 |
| Model 2*^d^* | 1.00 | 0.08 (0.01–0.63) | 0.20 (0.03–1.40) | 0.054 |
| Model 3*^e^* | 1.00 | 0.11 (0.01–1.27) | 0.27 (0.03–2.60) | 0.17 |
| Elevated TG |  |  |  |  |
| Crude | 1.00 | 1.00 (0.33–3.05) | 0.68 (0.46–1.03) | 0.23 |
| Model 1*^c^* | 1.00 | 1.28 (0.33–5.07) | 1.00 (0.19–5.18) | 0.93 |
| Model 2*^d^* | 1.00 | 1.26 (0.32–5.00) | 0.92 (0.18–4.83) | 0.99 |
| Model 3*^e^* | 1.00 | 3.05 (0.55–16.83) | 2.39 (0.30–19.01) | 0.29 |
| Reduced HDL-C |  |  |  |  |
| Crude | 1.00 | 0.36 (0.12–1.11) | 0.37 (0.12–1.11) | 0.06 |
| Model 1*^c^* | 1.00 | 0.39 (0.12–1.35) | 0.57 (0.16–1.98) | 0.27 |
| Model 2*^d^* | 1.00 | 0.35 (0.09–1.40) | 0.87 (0.21–3.60) | 0.59 |
| Model 3*^e^* | 1.00 | 0.55 (0.11–2.78) | 1.59 (0.28–8.97) | 0.77 |
| *^a^*Data are odds (95% confidence interval). sRAGE, soluble receptor of advanced glycation end-products; BP, blood pressure; FBG, fasting blood glucose; TG, triglyceride; HDL-C, high density lipoprotein cholesterol.  *^b^* Multiple logistic regression analysis.  *^c^* Adjusted for age (continuous variable), smoking status (never, former, or current), drinking status (never, ≤ 3 day(s)/week, 4–6 days/week, or every day), educational level (≥college or not), occupation (desk work or non-desk work), depressive symptoms (Self-Rating Depression Scale ≥40 or not), physical activity (<1.0 METs·hour/week, 1.0–22.9 METs·hour/week, or ≥23.0 METs·hour/week), total energy intake (continuous variable), sleep time (≥6 and ≤8 hours/day or not), and eGFR (continuous variable).  *^d^* Additionally adjusted for serum high sensitivity C-reactive protein concentration (continuous variable).  *^e^* Additionally adjusted for mutual metabolic syndrome components. | | | | |

| **TABLE S3**. Odds ratios of MetS risk factors by hsCRP and sRAGE categories in women (n = 176) *^a^*. | | | | | | | | |
| --- | --- | --- | --- | --- | --- | --- | --- | --- |
|  | Crude |  |  | Model 1*^c^* |  |  |  |  |
| MetS | Odds ratio (95%CI) | *P^b^* |  | Odds ratio (95%CI) | *P^b^* |  |  |  |
| Higher hsCRP/lower sRAGE (n =48) | 1 |  |  | 1 |  |  |  |  |
| Higher hCRP/higher sRAGE (n = 42) | 0.64 (0.21–1.94) | 0.43 |  | 0.62 (0.16–2.31) | 0.47 |  |  |  |
| Lower hsCRP/lower sRAGE (n = 40) | 0.72 (0.25–2.10) | 0.55 |  | 0.72 (0.19–2.68) | 0.62 |  |  |  |
| Lower hsCRP/higher sRAGE (n = 46) | 0.08 (0.10–0.63) | 0.017 |  | 0.04 (0.002–0.50) | 0.014 |  |  |  |
|  | Crude |  |  | Model 1 *^c^* |  |  | Model 4*^d^* |  |
| Central obesity | Odds ratio (95%CI) | *P^b^* |  | Odds ratio (95%CI) | *P^b^* |  | Odds ratio (95%CI) | *P^b^* |
| Higher hsCRP/lower sRAGE (n =48) | 1 |  |  | 1 |  |  | 1 |  |
| Higher hCRP/higher sRAGE (n = 42) | 0.43 (0.18–1.03) | 0.059 |  | 0.37 (0.14–0.98) | 0.046 |  | 0.49 (0.17–1.39) | 0.18 |
| Lower hsCRP/lower sRAGE (n = 40) | 0.33 (0.14–0.78) | 0.011 |  | 0.39 (0.15–1.04) | 0.060 |  | 0.47 (0.16–1.39) | 0.17 |
| Lower hsCRP/higher sRAGE (n = 46) | 0.10 (0.04–0.27) | <0.001 |  | 0.10 (0.04–0.30) | <0.001 |  | 0.15 (0.05–0.46) | 0.001 |
|  | Crude |  |  | Model 1 *^c^* |  |  | Model4*^d^* |  |
| Elevated BP | Odds ratio (95%CI) | *P^b^* |  | Odds ratio (95%CI) | *P^b^* |  | Odds ratio (95%CI) | *P^b^* |
| Higher hsCRP/lower sRAGE (n =48) | 1 |  |  | 1 |  |  | 1 |  |
| Higher hCRP/higher sRAGE (n = 42) | 0.68 (0.28–1.66) | 0.40 |  | 0.69 (0.26–1.83) | 0.45 |  | 0.97 (0.33–2.82) | 0.95 |
| Lower hsCRP/lower sRAGE (n = 40) | 0.97 (0.41–2.26) | 0.94 |  | 1.13 (0.43–2.96) | 0.80 |  | 1.55 (0.53–4.48) | 0.42 |
| Lower hsCRP/higher sRAGE (n = 46) | 0.33 (0.13–0.83) | 0.019 |  | 0.44 (0.16–1.21) | 0.11 |  | 0.74 (0.24–2.26) | 0.60 |
|  | Crude |  |  | Model 1 *^c^* |  |  | Model 4*^d^* |  |
| Elevated FBG | Odds ratio (95%CI) | *P^b^* |  | Odds ratio (95%CI) | *P^b^* |  | Odds ratio (95%CI) | *P^b^* |
| Higher hsCRP/lower sRAGE (n =48) | 1 |  |  | 1 |  |  | 1 |  |
| Higher hCRP/higher sRAGE (n = 42) | 0.74 (0.19–2.84) | 0.66 |  | 0.51 (0.08–3.16) | 0.47 |  | 0.44 (0.06–3.32) | 0.43 |
| Lower hsCRP/lower sRAGE (n = 40) | 1.11 (0.33–3.76) | 0.87 |  | 1.97 (0.36–10.70) | 0.43 |  | 0.95 (0.13–7.14) | 0.95 |
| Lower hsCRP/higher sRAGE (n = 46) | 0.15 (0.02–1.23) | 0.08 |  | 0.02 (0.00–0.52) | 0.02 |  | 0.01 (0.00–0.76) | 0.56 |
|  | Crude |  |  | Model 1 *^c^* |  |  | Model 4*^d^* |  |
| Elevated TG | Odds ratio (95%CI) | *P^b^* |  | Odds ratio (95%CI) | *P^b^* |  | Odds ratio (95%CI) | *P^b^* |
| Higher hsCRP/lower sRAGE (n =48) | 1 |  |  | 1 |  |  | 1 |  |
| Higher hCRP/higher sRAGE (n = 42) | 0.53 (0.15–1.91) | 0.33 |  | 0.55 (0.12–2.58) | 0.45 |  | 0.71 (0.12–4.15) | 0.70 |
| Lower hsCRP/lower sRAGE (n = 40) | 0.50 (0.14–1.80) | 0.29 |  | 0.35 (0.07–1.64) | 0.18 |  | 0.35 (0.06–2.01) | 0.24 |
| Lower hsCRP/higher sRAGE (n = 46) | 0.10 (0.01–0.84) | 0.034 |  | 0.09 (0.01–1.33) | 0.08 |  | 0.32 (0.02–6.01) | 0.45 |
|  | Crude |  |  | Model 1 *^c^* |  |  | Model 4*^d^* |  |
| Reduced HDL-C | Odds ratio (95%CI) | *P^b^* |  | Odds ratio (95%CI) | *P^b^* |  | Odds ratio (95%CI) | *P^b^* |
| Higher hsCRP/lower sRAGE (n =48) | 1 |  |  | 1 |  |  | 1 |  |
| Higher hCRP/higher sRAGE (n = 42) | 0.28 (0.08–0.95) | 0.041 |  | 0.33 (0.09–1.25) | 0.40 |  | 0.38 (0.09–1.67) | 0.20 |
| Lower hsCRP/lower sRAGE (n = 40) | 0.20 (0.05–0.74) | 0.017 |  | 0.19 (0.04–0.86) | 0.031 |  | 0.18 (0.03–1.06) | 0.06 |
| Lower hsCRP/higher sRAGE (n = 46) | 0.10 (0.02–0.52) | 0.005 |  | 0.13 (0.02–0.67) | 0.015 |  | 0.29 (0.05–1.86) | 0.19 |
| *^a^* Participants were categorized by the median values (1547.9 pg/mL for sRAGE; 0.23 mg/L for hsCRP). CI, confidential interval ; sRAGE, soluble receptor of advanced glycation end-products; BP, blood pressure; FBG, fasting blood glucose; TG, triglyceride; HDL-C, high density lipoprotein cholesterol.  *^b^* Multiple logistic regression analysis.  *^c^* Adjusted for age (continuous variable), smoking status (never, former, or current), drinking status (never, ≤ 3 day(s)/week, 4–6 days/week, or every day), educational level (≥college or not), occupation (desk work or non-desk work), depressive symptoms (Self-Rating Depression Scale ≥40 or not), physical activity (<1.0 METs·hour/week, 1.0–22.9 METs·hour/week, or ≥23.0 METs·hour/week), total energy intake (continuous variable), sleep time (≥6 and ≤8 hours/day or not), and eGFR (continuous variable).  *^d^* Additionally adjusted for mutual metabolic syndrome components. | | | | | | | | |
